# Strengthening life-course immunisation in migrant populations: access, equity, and inclusion

**DOI:** 10.1016/j.lanepe.2023.100806

**Published:** 2024-05-28

**Authors:** Felicity Knights, Jessica Carter, Anna Deal, Alison Crawshaw, Oumnia Bouaddi, Nuria Sanchez-Clemente, Farah Seedat, Sam Vanderslott, Rachel Eagan, Daphne E. Holt, Yusuf Ciftci, Miriam Orcutt, Holly Seale, Santino Severoni, Sally Hargreaves

**Affiliations:** a The Migrant Health Research Group, Institute for Infection and Immunity, St George's University of London, London, UK; b Coalition for Life-Course Immunisation (CLCI), UK; c International School of Public Health, Mohammed VI University of Sciences and Health, Casablanca, Morocco; d School of Population Health, University of New South Wales, Australia; e Oxford Vaccine Group, Department of Paediatrics, University of Oxford, Oxford, UK; f The Vaccine Confidence Project, LSHTM, London, UK; g The Health and Migration Programme, World Health Organization, Geneva, Switzerland; h The Lancet Migration European Regional Hub, UK; i Mohammed VI Center for Research and Innovation, Rabat, Morocco; j NIHR Oxford Biomedical Research Centre, Oxford, Oxfordshire, UK

**Keywords:** Migrant health, Life-course vaccination, Health inequalities, Catch-up vaccination, Vaccine acceptance and demand

## Abstract

Adult and adolescent migrants worldwide, and those arriving in Europe, are an under-immunised group for routine vaccinations due to missed childhood vaccines and doses in their countries of origin, and their subsequent marginalisation from health and vaccination systems. Declining population-level coverage for routine vaccines across Europe, which has accelerated post-pandemic, places these and other under-immunised populations at even greater risk of vaccine-preventable diseases. However, despite clear guidelines around the importance of delivering ‘catch-up’ vaccination throughout the life-course, migrants are rarely effectively incorporated into routine vaccination programmes on arrival to Europe. These populations have subsequently been involved in outbreaks, including measles and diphtheria, and are missing opportunities to receive more recently introduced vaccines such as HPV to align them with European vaccine schedules. WHO's new Immunization Agenda 2030 places a renewed emphasis on equitable access to vaccine systems and integrating catch-up vaccination for missed vaccines and doses throughout the life-course. In addition, lessons learned and innovations from the COVID-19 pandemic merit further consideration in the design and delivery of more inclusive vaccination programmes. We describe current gaps in policy and practice around life-course vaccination in migrant populations, key factors that drive low vaccine uptake and coverage, and explore the benefits of participatory approaches to designing and delivering interventions with impacted communities, to define new strategies to advance vaccine equity across the Region.

## Introduction

Adult and adolescent migrants in Europe—particularly migrants arriving from low-income and middle-income countries—are at high risk of under-immunisation for routine vaccinations resulting from missed vaccines and vaccine doses as children, and their marginalisation from health and vaccination systems. Migrants face many barriers in receiving a full vaccination schedule as children, adolescents, and adults including the disruption of vaccine systems in their countries of origin due to war, poor infrastructure, vaccine shortages, as well as personal, socio-structural and physical barriers including their inherent mobility. In addition, vaccination schedules in their home country may differ, missing, for example, more recently added vaccines such as human papillomavirus vaccine (HPV), which may form part of schedules in migrants' countries of origin. Greater emphasis should therefore be placed on aligning these populations with European vaccination schedules on or soon after arrival so that opportunities for primary prevention of diseases are not missed.^1^, ^2^, ^3^, ^4^ In a large recent analysis of refugees coming to the UK via a government resettlement programme, only 11% were fully aligned with the UK schedule for polio, 34% for measles, and 5% for diphtheria and tetanus, with adults more likely than children to be under-immunised.^5^ Migrants in European countries, along with other under-immunised groups, have been involved in outbreaks of vaccine-preventable diseases (VPDs)^6^ (see Panel 1). A recent outbreak of diphtheria in asylum accommodation in the UK further reinforces the importance of engaging adolescents and adults in ‘catch-up’ vaccines on arrival for vaccines, doses, and boosters they may have missed in their home countries as children.^8^

*Panel 1* **Coverage for routine immunisations and outbreaks involving migrants.**

Migrants have been involved in vaccine-preventable disease (VPD) outbreaks in Europe, with a disproportionate burden of outbreaks (particularly of measles, varicella and hepatitis A) amongst adult and child migrants living in temporary shelters or camps, alongside specific nationality groups.^6^^,^^7^ 47% of outbreaks in one European study were reported from temporary settlements or refugee camps, which have become an increasingly important feature in Europe as the numbers of forced migrants residing in such conditions continues to increase since 2015.^6^ Poor sanitation and overcrowding makes these settlements highly conducive to experiencing outbreaks.^8^^,^^9^

Several major measles outbreaks were recorded in Europe during 2015 and 2020, with over 60,000 cases and 98 deaths reported, involving under-vaccinated adult migrants.^6^ Major clusters in Greece, Belgium and Germany during 2019 suggested that adult migrants from Eastern European countries may be involved.

The European Centre for Disease Prevention and Control (ECDC) has also reported a recent increase in diphtheria cases, mostly among asylum seekers, with 153 cases reported by eight European countries in 2022 among migrants, resulting in one death.^10^ The UK has also seen a major diphtheria outbreak among asylum seekers housed in reception centres being found positive for diphtheria in 2022.^8^^,^^11^

Coverage for routine vaccinations among arriving migrants is low, with multiple studies confirming this (Hargreaves S, unpublished data).^3^ A meta-analysis of 75,089 migrants residing in 12 EU/EEA countries highlights that pooled immunity coverage among migrant populations was below the recommended herd immunity threshold (HIT) target for diphtheria (n = 7, 57.36% [95% CI 43.05–71.66%] I^2^ = 99% vs HIT 83–86%), measles (n = 21, 83.7% [95% CI: 79.18–88.21] I^2^ = 99% vs HIT 93–95%), and mumps (n = 8, 67.1% [95% CI: 50.6–83.6] I^2^ = 99% vs HIT 88–93%), and midway for rubella of 85.6% (n = 29, 95% [CI: 83.1–88.1%] I^2^ = 99% vs HIT 83–94%) (Hargreaves S, unpublished data).

Unfortunately, adolescent and adult migrants are rarely considered in vaccination programmes on arrival to European countries,^12^ with the focus being predominantly on very young children and in some cases specific migrant groups such as refugees.^2^ This is compounded by the fact that in many European countries there are shortfalls in vaccine programmes targeting adolescents and adults in the general population, with calls for greater focus on life-course immunisation for all (https://www.cl-ci.org). This has resulted in a growing under-immunised adult population group who could be targeted by specific vaccination initiatives in order to improve vaccine coverage for certain key infections. The COVID-19 pandemic highlighted numerous challenges to integrating migrants in vaccination programmes, as well as other groups who were found to be structurally marginalised from health and vaccine systems during the pandemic. Yet the pandemic also prompted innovations and opportunities for more inclusive service delivery and policymaking in migrant health. As European countries place renewed efforts on improving declining vaccine coverage for routine vaccinations, the innovations must be seized upon to bolster these efforts.^12^^,^^13^ In this Viewpoint, therefore, we aim to describe the current status quo regarding life-course vaccination in migrant populations, with a specific focus on policy and practice in European countries. International migrants to Europe are a diverse group, including refugees, asylum seekers, undocumented migrants, international students, and labour migrants, with varying social determinants of health and reasons for migration which affects their access to, perceptions of, and decision-making regarding vaccinations.

## Recommendations vs reality in Europe

The WHO's new Immunization Agenda 2030 (IA2030),^14^ and subsequent WHO reports^15^^,^^16^ have placed a renewed focus on achieving equitable access to vaccines for marginalised populations including migrants, and integrating catch-up vaccination for missed vaccines and doses throughout the life course. WHO recommends that it is always ‘better to vaccinate late than never’ and stresses the importance of countries defining a catch-up vaccination policy and catch-up vaccination schedule,^17^ to close immunisation gaps that compound as populations increase in age. Indeed, for most vaccine-preventable diseases (VPDs), providing vaccines late still offers protection against morbidity and mortality, as well as reducing transmission and risk of outbreaks.

Specific WHO guidance for catch-up vaccination is available.^18^ In Europe, the European Centre for Disease Prevention and Control (ECDC) has recently published guidance on catch-up vaccination for adult, adolescent, and child migrants arriving to European countries,^19^^,^^20^ requesting that healthcare providers consider revaccinating adult migrants with uncertain vaccination status or no recorded history of vaccination for measles, mumps, rubella and diphtheria, tetanus, and polio (see Panel 2). It is very common for migrants to have no vaccine records at all on presentation to a European health service, because they were not in the host country as children when most vaccinations are administered, which presents major challenges to health systems and front-line health workers who are often unclear as to how to respond. A systematic review found that targeting migrants for catch-up vaccination is cost effective for presumptive vaccination for diphtheria, tetanus, and polio, and there was no evidence of benefit of carrying out pre-vaccination serological testing in this group.^21^ Additional travel vaccines may be recommended if they return to their home country, or for specific occupations or at-risk groups (eg, tetanus and hepatitis B vaccines for health and care workers), but little research to date has explored uptake and delivery of occupational vaccines in the growing number of migrants coming to Europe to work. Europe now has the highest number of migrant workers globally, a 24% share of the migrant workforce.^22^ WHO's recent European Immunization Agenda 2030^23^ specifically calls for States to ensure all groups have equitable access to vaccine services and to identify and offer vaccination to all people who have missed vaccinations.

*Panel 2* **European Centre for Disease Prevention and Control guidance on catch-up vaccination in child, adolescent, and adult migrants on arrival to Europe (Reproduced from19 with permission).**

√ Offer vaccination against measles/mumps/rubella (MMR) to all migrant children and adolescents without immunisation records as a priority.
√ Offer vaccination to all migrant adults without immunisation records with either one dose of MMR or in accordance with the MMR immunisation schedule of the host country.
√ Offer vaccination against diphtheria, tetanus, pertussis, polio and HiB (DTaP-IPV-Hib) to all migrant children and adolescents without immunisation records as a priority.
√ Offer vaccination to all adult migrants without immunisation records in accordance with the immunisation schedule of the host country. If this is not possible, adult migrants should be given a primary series of diphtheria, tetanus, and polio vaccines.
√ Offer hepatitis B vaccination series to all migrant children and adolescents from intermediate/high prevalence countries (≥2%–≥5% HBsAg) who do not have evidence of vaccination or immunity.

Globally, the focus for strengthening vaccine programmes is on ‘zero dose children’—children who have failed to receive any routine vaccinations—a phenomenon that increased during the COVID-19 pandemic as a result of a global disruption to vaccination services, resulting in increasing global morbidity and mortality from VPDs.^24^ Data show that 18 million children in 2021 did not receive a single dose of vaccines, with 60% living in just 10 countries—Angola, Brazil, Democratic Republic of the Congo, Ethiopia, India, Indonesia, Nigeria, Pakistan, Philippines and Mexico.^17^^,^^24^ Understanding where the gaps in global vaccination coverage lie is imperative as this will help inform catch-up strategies for older groups. Ultimately, strengthening immunisation delivery across the whole life course is needed to prevent zero dose children becoming, zero dose adults. One European study identified 23 significant determinants of under-immunisation in migrants in Europe (p < 0.05), including African origin, recent migration, and being a refugee or asylum seeker,^25^ but more work is needed in this area to guide healthcare providers and planners to target and tailor catch-up vaccination programmes.

Unfortunately, European health systems have yet to rise to the challenge. In a survey of EU/EEA countries, vaccine availability and recommendations to vaccinate migrant children, adolescents and adults were found to vary considerably, with only 15 EU/EEA countries offering limited vaccines—prioritising diphtheria-tetanus, measles-mumps-rubella and polio vaccinations.^26^ In a comparative policy analysis only 4 (12.5%) of 32 EU/EEA countries had specific policies on vaccination of adult migrants,^27^ with 19% focused around catch-up vaccination in response to a specific outbreak,^27^ and several European countries reporting charging migrants' fees for routine vaccinations.^12^ Pre-COVID, only one European country (out of the 6 included), had standard procedures to guarantee the migrants’ access to vaccination at the community level. In this case, this involved dedicated social workers accompanying migrants to health facilities for vaccination.^28^ During the COVID-19 pandemic, WHO raised concerns that several countries did not initially include migrants in national plans when vaccines became available.^17^ An analysis of national deployment and vaccination plans submitted to the COVAX Facility (Feb–March 2021) reported that most countries did not explicitly include migrants (72%); just over half explicitly included refugees and asylum seekers (53% of 64 countries that have more than 500 refugees); and only 17% explicitly included irregular migrants.

### Access and uptake barriers on arrival to Europe

Beyond these obvious policy limitations, research has identified multiple access and uptake barriers in these populations which will need to be better considered in order to improve coverage in these groups.^1^^,^^3^^,^^29^ The COVID-19 pandemic provided a stark reminder of vaccine inequity in Europe, and the difficulties public health teams faced when reaching out to migrant and ethnic minority communities. A large UK study of 465,470 migrants, for example, found slower uptake of the first dose of the COVID-19 vaccine across all age-groups for migrants compared with the general population, with this population more likely to have delayed uptake of COVID-19 vaccines and to not have received their second or third dose,^30^ a finding mirrored in other European countries.^1^^,^^31^

WHO's Behavioural and Social Drivers of Vaccination (BeSD) framework,^32^ which brings together several existing models and frameworks to conceptualise drivers of under-immunisation, emphasises three core domains that may affect uptake: what people think and feel about vaccines; social processes that drive or inhibit vaccination (which combine to influence an individual's motivation or hesitancy to seek vaccination); and practical factors (such as a migrants lack of entitlement to receive free health care, and service barriers such as lack of specific guidelines and knowledge of health-care professionals).^33^ Research has identified multiple issues within each of these domains in relation to migrants in the European context^1^^,^^29^^,^^34^: including distrust of health system or authorities, stigma around specific vaccines, migrants' sense of alienation and disempowerment, and practical factors such as resource and capacity constraints, logistics issues, misinformation circulating on social media, or lack of information.^35^

Many studies describe well-known barriers in the provision of healthcare services for migrants, including language barriers, cultural and communication barriers, and low levels of literacy, combined with a lack of experience and knowledge around a host country's health system amongst migrant patients.^36^^,^^37^ In addition, refugees, asylum-seekers, and those residing in closed settings such as camps, detention and reception centres and informal settlements, as well as specific excluded groups such as irregular migrants and Roma communities may be also disproportionately impacted by practical and legal barriers to health systems, as well as higher levels of stigma, discrimination and marginalisation that can in turn lead to mistrust of public health and vaccination services. Data show a range of service-level barriers, including difficulties with navigating health systems, challenges registering with general practice, variations in models of care, policy and screening practices across countries, lack of written health records (associated with increasing age/lowest for refugees from sub-Saharan Africa),^38^ and cultural exclusion.^39^ The lack of familiarity with the health care system often entails lack of information regarding the logistics to get to health services among newly arrived refugees or a lack of understanding of the function of a primary health care system.^40^ A key issue that has been demonstrated to affect the quality of consultations is the use of professional interpreters by clinicians.^41^

While the ECDC recommends offering catch-up vaccination in the absence of reliable documentation and vaccination records, one study in France found that GPs rely on the information provided by migrant patients, and may also be reluctant to revaccinate due to concerns about “hyperimmunization”,^42^ lack of appropriate training, or insufficient knowledge. Health care providers also raise the issues of inadequacy of resources (particularly human and time related) and the conflict and contradiction between professional ethics, aiming to protect the interests of patients, and legal mandates that tend to restrict the right to health of migrants. Where attempts have been made to tackle life-course immunisation in mobile groups, research has highlighted just how difficult it is to implement such programmes, with lack of time and resources to do catch-up vaccination cited as key concerns.^37^

#### Learning from COVID-19

The COVID-19 pandemic has presented a range of new opportunities and innovations in vaccine service delivery and policymaking to these groups, which merit greater consideration going beyond the pandemic.^13^^,^^43^ In particular, the pandemic has provided some insights into facilitators of vaccine uptake in migrant groups and highlighted the extent to which these populations have traditionally been overlooked and excluded from health decision-making.^9^^,^^44^ On a policy level, for example, several European governments guaranteed irregular migrants equal access to healthcare and the vaccination during the pandemic in an attempt to increase coverage.^16^^,^^45^^,^^46^ Learning from the mass vaccination campaigns for COVID-19, and their limited success at engaging marginalised and migrant groups (who showed slower and lower levels of uptake), has emphasised the need for new thinking around adapting vaccine initiatives to migrants to ensure their inclusion, and to avoid generalising migrants as a single, homogeneous group. More inclusive and accessible services and policies, including using non-clinical, familiar and community-based settings, culturally tailored and community-led information campaigns and interventions, and mechanisms to offset costs need exploring now for routine immunisations.

There is increased recognition of the need for meaningful participation and active involvement of migrants as equal partners in the research process, so that interventions are informed by contextually-specific understanding of these groups' needs and lived experiences and implemented and led by community members.^47^^,^^48^ This perspective has also been emphasised by The Regional Risk Communication and Community Engagement (RCCE) Interagency Working Group's proposed response to reduce the negative impacts of COVID-19, which sets out the importance of ensuring that both strategies and implementation approaches are *‘community-led, data-driven, collaborative, and reinforce capacity and local solutions’*.^49^ There is the potential for participatory research to bring about more relevant and sustainable vaccination services with better use of time and resources and ensure effective tailoring of health information, services, and interventions to different migrant communities.^44^^,^^50^ Most research to date has not considered the views of migrant communities themselves and is instead driven largely by the interests of academics, policymakers and clinicians.^51^ This should be better considered from design to delivery and through to evaluation, and where possible enable and empower communities to lead in bringing about their own solutions to improving vaccination uptake and reducing the risk of future outbreaks. This is now a prime focus of WHO EURO, who are developing training specifically around conducting participatory health research with migrant communities and have released a country implementation guide regarding migrants' involvement in research.^51^ They propose that policy changes are needed within national contexts to achieve this, involving increased involvement of migrants in policy development, service delivery, and research; availability of funding that reflects the unique demands and approaches of participatory health research; and development of further, specific training to ensure all stakeholders are equipped to involve migrants meaningfully in participatory health research.

**Fig. 1:**
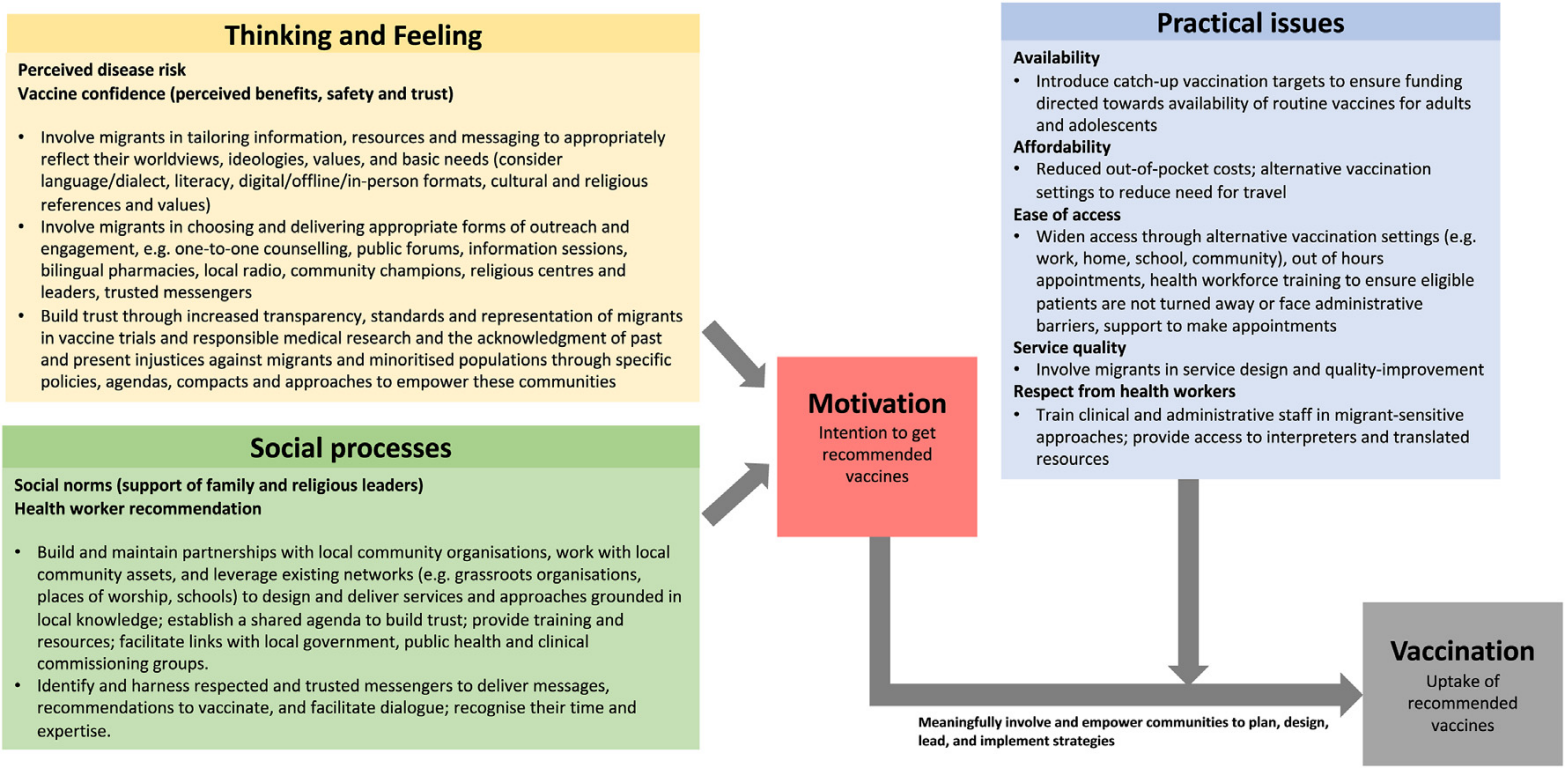
**Strengthening uptake of routine immunisations in migrant communities: Strategies to consider based on the behavioural and social drivers (BeSD) of vaccination framework (Based on Brewer et al.**^32^**)**.

We highlight key considerations for working with migrant communities to increase awareness and uptake in Fig. 1.

### Next steps in policy and practice

It is now critical that we start working towards a unified commitment to life-course vaccination of migrants. Declining population-level coverage for routine immunisations across Europe, which has accelerated post-pandemic, places these and other under-immunised populations at even greater risk.

The development of unified European guidelines is unlikely to occur, given variations in migration patterns, resources, and political commitment. Rather, individual countries should be prompted to develop (or strengthen) their national guidelines, and practices should be homogenised within the country to ensure effective catch-up vaccination delivery. This should include a focus on the strengthening of research and routine data collection on vaccine uptake by ethnicity and country of origin, the enhancement of healthcare services to ensure they are culturally responsive, alongside development of migrant-specific guidance and training packages for healthcare providers with a focus on catch up vaccination.^52^ Beyond guidance and training, investment in new service models, considering what information is required in what format, where vaccines can be offered, and by who, alongside implementation of catch-up calculators and digital innovations to support clinical decision making^37^ may facilitate more equitable vaccine provision. Central to success will be meaningful engagement with at-risk communities and the co-production of tailored and targeted programmes to drive uptake, to raise awareness of the benefits of vaccinations across the life course, tackle misconceptions, and ensure they have the knowledge and resources to advocate for their own health and that of their communities. Panel 3 outlines key areas for policy, practice, and research going forward.

*Panel 3* **Strengthening life-course immunisation in migrant populations: policy, practice, and research.**

**Policy**

√ Ensure migrants of all ages are incorporated into policies, strategies, and initiatives relating to catch-up initiatives for routine immunisations, including newer vaccines such as HPV, occupational vaccines, and vaccines targeted towards at-risk groups eg, Hepatitis B. Specifically include migrant sub-groups who face barriers and exclusion from mainstream health systems—including undocumented migrants, asylum seekers/refugees, and those residing in camps and detention facilities and equip migrants communities with knowledge of their entitlements to vaccination systems on arrival.
√ Adequately fund and resource health systems so they can more effectively delivery catch-up vaccination to marginalised groups, given the additional time and resources this requires. Incentivise this through developing catch-up vaccination targets and funding pools.
√ Strengthen approaches to recording and collecting data on vaccine acceptance and demand in migrants presenting to European health systems to generate a stronger evidence-base to meaningfully inform policy and practice and improve knowledge translation of existing evidence into policy and practice.
√ Reduce access barriers by ensuring all routine vaccinations are free of charge and work multisectorally to promote inclusive policies that remove practical and legal barriers to vaccine services.

**Practice**

√ Train and support healthcare staff in understanding and delivering life-course vaccination in migrant groups and those with uncertain or incomplete vaccination status.
√ Recognise the importance of co-designing vaccine interventions with migrant communities to ensure that they are tailored to the diverse needs of migrant sub-groups and build trust and confidence in vaccines through continuous, inclusive engagement. Finance and support community-led interventions and trusted, respected community-based organisations and champions to represent and encourage engagement with vaccination initiatives.
√ Explore new migrant-inclusive models of vaccine service delivery, and innovations in service delivery models seen during the COVID-19 pandemic, including outreach, out of hours services, and delivery of vaccines in faith-based venues, community-based venues and trusted locations. Develop specific vaccination campaigns to provide culturally and linguistically tailored materials about routine immunisations alongside qualified interpreters
√ Work directly with migrant communities to draw on local knowledge and co-develop tailored strategies and vaccination messaging that effectively reach the desired audience and address context-specific barriers, misconceptions or misinformation where it exists.

**Research**

√ Undertake national data collection and analysis on routine vaccine uptake (disaggregated by migrant status, country of origin, age and gender) to capture vaccination coverage data for refugees and migrants, and assess vaccine uptake, inequalities between migrant sub-groups, and the impact of local or national immunisation initiatives.
√ Develop robust, large-scale qualitative and quantitative studies across migrant groups to identify the drivers of under-immunisation and vaccine hesitancy, to guide targeting of vaccination programmes, and undertake intervention design research to identify intervention points.
√ Acknowledge the historic injustices and misrepresentation of minority groups in medical research and develop institutional policies committed to improved transparency, representation and involvement which should be discussed and promoted more widely to build trust.
√ Support research into the lived experiences and vaccination needs and barriers of particularly vulnerable or underserved migrant groups such as migrants in camps, reception and detention centres, homeless shelters and other high-risk settings for VPD.
√ Invest in participatory and co-produced research studies and initiatives that share power with migrant groups involve relevant community stakeholders, and lead to co-produced, community-drive interventions. This will require funding bodies to adapt their requirements, criteria, and timelines to enable more community based and community-led research.

The impact of the COVID-19 pandemic on vaccine coverage across populations will be felt for years to come, putting under-vaccinated populations at increased risk. Increases in zero-dose children and dips in routine coverage will require targeted programmes and dedicated funding to rectify. It is important that we don't lose focus on moving towards enhancing vaccine coverage for older groups, including adult and adolescent migrants to the European Region. Central to this will be a much-needed change in policy and perspective, away from host countries seeing healthcare and vaccination systems as a privilege that new arrivals have not merited, to viewing healthcare provision as not only a human right but also an essential and cost-effective approach to improving both individual, community, and population-level health.

## Contributors

SH conceived the idea, with FK, JC, AC, and AD, who also led the writing of specific sections. All authors reviewed and revised the first draft and approved the final version.

## Declaration of interests

All authors declare no conflicts of interest.
